# The impact of regional disparities on the availability of meningococcal vaccines in the US

**DOI:** 10.1186/s12889-024-19081-w

**Published:** 2024-07-03

**Authors:** Katharina Schley, Eva Jodar, Jessica V. Presa, Sarah J. Willis, Christopher G. Prener

**Affiliations:** 1 Pfizer Pharma GmbH, Berlin, Germany; 2 Pfizer Inc, New York, NY USA; 3 Pfizer Inc, Collegeville, PA USA; 4 Pfizer Inc, Cambridge, MA USA

**Keywords:** Social equity, Meningococcal vaccination, Shared clinical decision making, Invasive meningococcal disease, Inequality, Vaccine access, Access barriers

## Abstract

**Background:**

In the United States (US), three types of vaccines are available to prevent invasive meningococcal disease (IMD), a severe and potentially fatal infection: quadrivalent conjugate vaccines against serogroups A, C, W, Y (MenACWY), and monovalent vaccines against serogroup B (MenB) as well as a newly licensed pentavalent vaccine (MenABCWY) protecting against serogroup A, B, C, W, and Y. The CDC’s Advisory Committee on Immunization Practices (ACIP) routinely recommends MenACWY vaccine for all 11- to 12-year-olds with a booster dose at 16 years. MenB vaccination is recommended based on shared clinical decision-making (SCDM) for 16- to 23-year-olds. Recently, the pentavalent meningococcal vaccine (MenABCWY) was recommended by the ACIP. Meningococcal vaccine uptake is suboptimal across the country, particularly among individuals with lower socioeconomic status (SES), despite these recommendations. The objective of the spatial analyses was to assess the relationship between stocking of MenACWY and MenB vaccines, area-level SES, and state-level policies.

**Methods:**

The number of MenACWY and MenB doses stocked by vaccinators was obtained from IQVIA and the CDC’s Vaccine for Children (VFC) program and compiled into a county-level dataset from 2016 to 2019. SES, as measured using the CDC’s Social Vulnerability Index (SVI), state-level school recommendations, and universal purchasing programs were among the main county-level covariates included to control for factors likely influencing stocking. Data were stratified by public and private market. Bayesian spatial regression models were developed to quantify the variations in rates of stocking and the relative rates of stocking of both vaccines.

**Results:**

After accounting for county-level characteristics, lower SES counties tended to have fewer doses of MenB relative to MenACWY on both public and private markets. Lower SES counties tended to have more supply of public vs. private doses. Universal purchasing programs had a strong effect on the markets for both vaccines shifting nearly all doses to the public market. School vaccination strategy was key for improving stocking rates.

**Conclusions:**

Overall, the results show that MenACWY has greater stock relative to MenB across the US. This difference is exacerbated in vulnerable areas without school entry requirements for vaccination and results in inequity of vaccine availability. Beyond state-level policy and SES differences, SCDM recommendations may be a contributing factor, although this was not directly assessed by our model.

**Supplementary Information:**

The online version contains supplementary material available at 10.1186/s12889-024-19081-w.

## Introduction

*Neisseria meningitidis* is a bacterium that causes a severe and potentially deadly infection with rapid onset called invasive meningococcal disease (IMD) [1]. Meningococcal bacteria are transmitted by exchanging respiratory secretions or droplets, and person-to-person interactions with either infected or asymptomatic carriers [2]. IMD incidence is highest in children < 1 year old, with a second peak in adolescents and young adults. In 2019, before the COVID-19 pandemic, the incidence of IMD in the US was 0.82 per 100,000 people among infants aged < 1 year, 0.04 cases per 100,000 people among adolescents aged 11 to 15 years, and 0.13 per 100,000 among persons aged 16 to 23 years [3]. The case fatality ratio reported by the Centers for Disease Control and Prevention (CDC) is 15.1 deaths per 100 cases [4]; internationally, fatality rates range from 4.1 to 20% [5]. Sequelae from IMD may be physical, emotional, and neurological including amputations, anxiety, and hearing loss in about 25% of survivors [6].

In the US, three types of vaccines are available—quadrivalent conjugate vaccines against serogroups A, C, W, Y (MenACWY) and monovalent vaccines against serogroup B (MenB) as well as a newly licensed pentavalent vaccine (MenABCWY) protecting against serogroup A, B, C, W, and Y. The CDC’s Advisory Committee on Immunization Practices (ACIP) routinely recommends MenACWY vaccine for all adolescents aged 11 to 12 years with a booster dose at 16 years. MenB vaccination is recommended for those aged 16 to 23 years (2 doses) based on shared clinical decision-making (SCDM), which is based on an individual risk assessment and the vaccination decision should be informed by a discussion between health care provider and patient [2, 7, 8]. ACIP recently recommended a MenABCWY vaccine when both MenACWY and MenB are indicated during the same visit [9]. Based on the most recent data, uptake of ≥ 1 dose of MenACWY is 88.6% among adolescents aged 13 to 17 years; with 60% of adolescents receiving ≥ 2 doses [10]. For MenB, uptake of ≥ 1 dose was 29.4% among adolescents aged 17 years [11]. Additionally, vaccine uptake varies by state, for example, uptake of ≥ 1 dose of MenACWY vaccine varies from 55.5% in Mississippi to 97.9% in Iowa [10]. Vaccine school requirements vary from state to state which might play a role in driving these differences across states as well as state purchasing policies.

Health insurance coverage plays a crucial role in determining access to health care services, including vaccines, as do disparities in race/ethnicity, geography, and socioeconomics. A recent systematic literature review assessing the impact of social determinants of health, specifically for meningococcal vaccination, showed a consistent variance in MenACWY and MenB coverage across population subgroups [12]. ACIP recognizes the importance of considering issues related to health equity and includes this as a domain in the ACIP’s Evidence to Recommendations (EtR) framework [13].

To our knowledge, no evidence in the US exists analyzing the relationship between the availability of MenACWY and MenB and regional indicators like socioeconomic status (SES), presence of local school recommendations, number of physicians, and level of rurality. To fill this gap, we designed a study to (1) assess if there are disparities in vaccine availability by county-level SES and state level policies (universal purchase programs and school vaccination recommendations), stratified by public and private delivery of vaccines; (2) estimate the relationship between regional disparities and stocking of meningococcal vaccines; and (3) evaluate the relationship of differences in regional characteristics to the availability for MenACWY vs. MenB vaccines across the US.

## Methods

### Study design

This was a cross-sectional, ecological study with data aggregated at the US county level. All counties with valid data (non-missing observations for any variable included in our final models) in the US – the 48 states including Alaska and Hawaii, and the District of Columbia – were eligible for inclusion. American overseas territories (American Samoa, Guam, Northern Mariana Islands, Puerto Rico, and the US Virgin Islands) were not included because of limited data availability and/or the lack of county equivalents. Counties with missing data on any of the variables described in “Data sources” section were also excluded.

### Data sources

#### Vaccine stocking data

Stocking of meningococcal vaccines is used as a proxy to describe access to meningococcal vaccines. Our approach aligns with the approach in Rodriguez Santana et al. (2023) [14] which describes the opportunity for patients to use health care services. The authors use preventive services and vaccination as an example of these concepts where supply meets the patient’s need but not their demand.

Stocking was measured as cumulative gross deliveries by type of vaccine and provider per 1,000 adolescents aged between 10 and 19 years for 2016 to 2019. Both the commercial and VFC stocking data were provided to the study team by IQVIA. IQVIA Drug Distribution data (DDD) delivery data, containing sales outlet delivery information including a ZIP5, were utilized to locate those ZIP Codes within the best match county. In cases where ZIP Codes crossed county boundaries, outlets were allocated based on the share of the ZIP Code with a higher percentage of commercial addresses, as determined by data from the US Department of Housing and Urban Development. Counties with no recorded deliveries were assigned a 0 based on guidance from IQVIA.

#### Social vulnerability index (SVI)

To measure county-level socioeconomic disparities, we have used the Social Vulnerability Index (SVI), a quantitative measure to assess socioeconomic differences in threats and recovery from hazards and natural disasters based on 15 social factors, which include poverty, lack of vehicle access, minority ethnicity, and crowded housing (Sect. 1, Table S2 of the Supplemental Material) [15]. SVI combines social, cultural, and economic factors contributing towards disparity in one index [16]. The SVI scores were calculated using the 2015 to 2019 5-year American Community Survey (ACS) using an R script that replicates the CDC’s methodology from their 2018 data release [17]. SVI has been used in other studies as a measure for social vulnerability to understand health disparities [18].

#### Census data

All Census Bureau data were accessed via the Census Bureau’s API using the R package *tidycensus*. The ACS was the preferred data source as it captures SES data, health insurance status, and other variables related to health and the provision of health care services helpful for the analysis. The 2015–2019 5-year ACS estimates were used as they offered more precise point estimates than the single-year ACS estimates, and this time period offered the best overlap with the study period.

#### State vaccination requirements

Data on meningococcal state requirements were acquired from each state’s Department of Health or Department of Education’s official webpage. Each state has a list of required immunizations to attend school. The requirements were either listed by age or by school grade. Due to the mobility of students for college/university attendance and the challenges associated with attributing the vaccination to the right location, college/university requirements were out-of-scope of this analysis.

To classify the meningococcal requirements, the required immunizations for seventh grade (10-, 11-, 12-year-olds) and eleventh or twelfth grade (16 + year olds) were examined. The requirement was subsequently classified as:

- “Required”: the state lists a meningococcal vaccine as a required vaccine to enter school.
- “Recommended”: the state explicitly recommends the vaccine on their school immunization requirements page but does not require it for school entry.
- “No”: the state neither requires nor recommends any type of meningococcal vaccination for school entry.

State-specific requirements can be found in Sect. 1, Table S3 of the Supplemental Material.

**Fig. 1:**
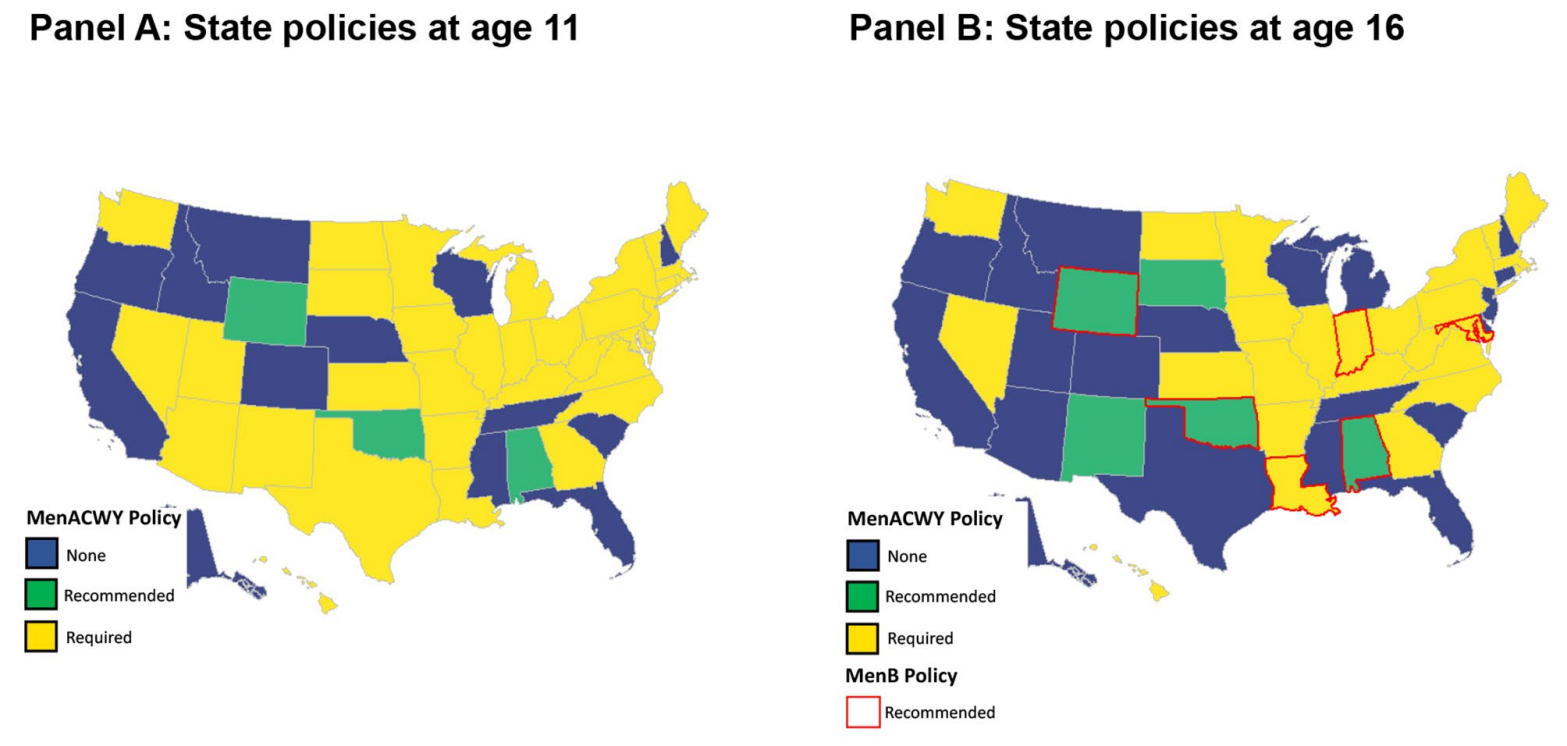
School Requirements and recommendations for vaccination against MenACWY and MenB at age 11 and 16

A total of 36 states in the US require a MenACWY vaccination at age 11 for school attendance, and 3 states recommend but do not require the vaccine for school attendance. In total, 13 states have no MenACWY vaccination recommendation or requirement in place for school attendance at age 11 (Fig. 1A). For the recommended booster dose at age 16, fewer states require or recommend MenACWY vaccination for school attendance. Only 6 states had a recommendation for MenB vaccination for school attendance at age 16 (Fig. 1B).

**Fig. 2:**
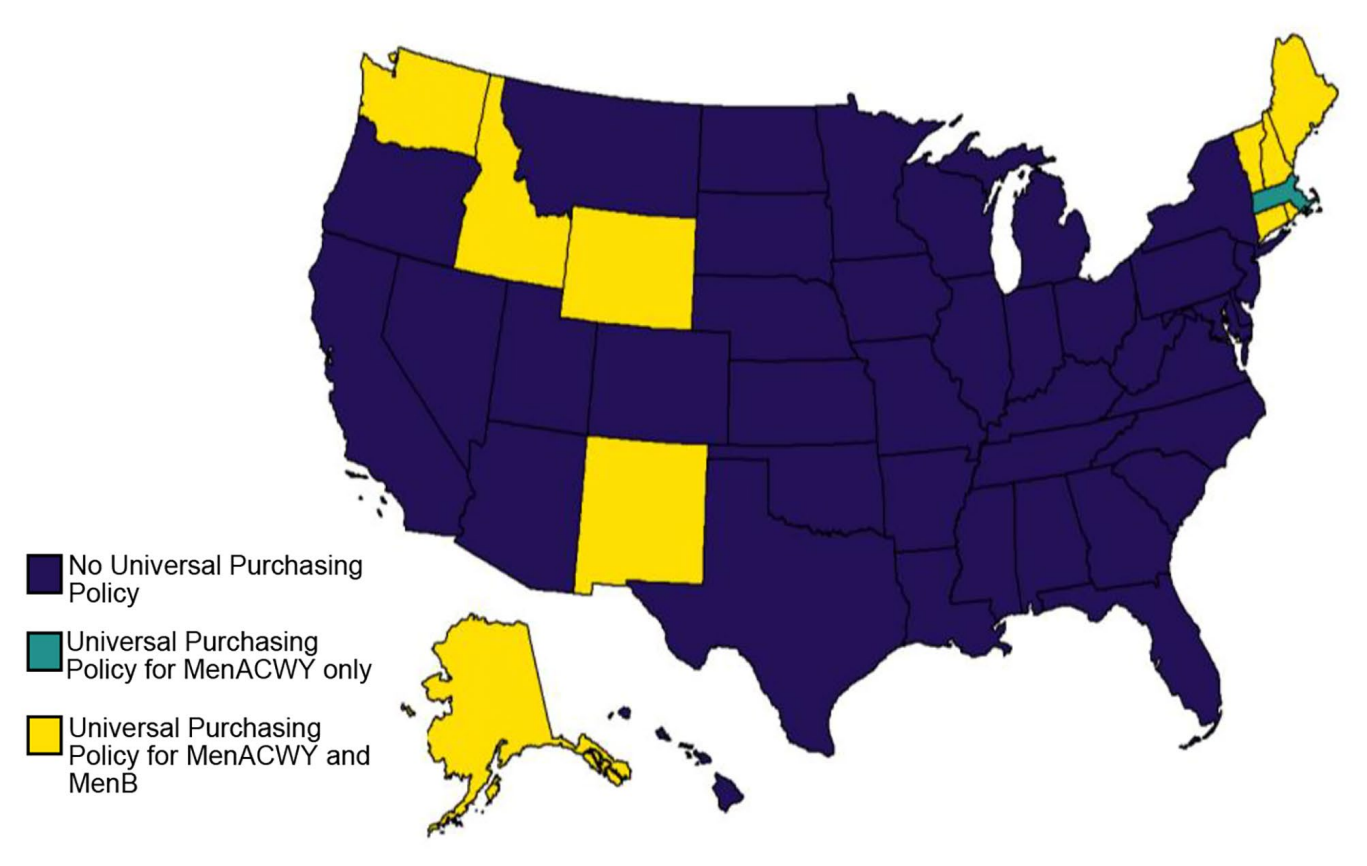
Universal purchasing policies for MenACWY and MenB

#### Universal purchasing policies

In some states, vaccines are purchased centrally by the state government and supplied to all children in the state regardless of their insurance status – this is referred to as a universal purchasing program [19]. Currently 10 states offer a universal purchase program for both MenACWY and MenB vaccines including Alaska, Connecticut, Idaho, Maine, New Hampshire, New Mexico, Rhode Island, Vermont, Washington, and Wyoming (Pfizer subject matter experts). In addition, Massachusetts offers MenACWY vaccines on a universal purchasing program basis (Fig. 2).

Overall, it is worth noting that all vaccines recommended by the ACIP to infant, young children, and adolescent are covered by the VFC program, ensuring access to vaccinations for minors from families unable to afford them [20, 21]. Further information on the VFC program can be found in the Supplemental Material.

#### Other data

Several data sources were included to control for additional factors influencing the regional stocking of vaccines. Data on the presence of military medical centers (what we label as “major facilities”) and basic training sites from the Department of Defense (DoD) were included as MenACWY is required for recruits starting basic military training [22]. Rurality, number of health care providers, and tribal lands and Indian Health Service facilities were also captured. Refer to Sect. 1, Table S1 of the Supplemental Material for a full list of variables included and additional details on data sources.

### Statistical methods

#### Hierarchical bayesian spatial regression models

A series of regression models were developed to quantify variations in stocking of MenB and MenACWY vaccines at the county level across the US while accounting for spatial correlation in the outcomes. The main association of interest was between rates of vaccine stocking and both SES level of the county (as measured with the SVI SES theme) and state level policies. These models were used to assess the proportion of MenACWY vs. overall meningococcal doses, including MenB, overall and on the public and private markets. Separate logistic regressions were also fit for estimating the total number of MenACWY or MenB stocked in a county on the public market. The denominator was the total number of doses (public and private). Finally, separate negative binomial regression models were fit for each vaccine and by public/private doses to estimate the per capita rate of stocking. The outcome was the number of doses distributed to HCPs in the county in 2016 to 2019. An offset (denominator) was included to adjust for the size of the eligible population aged 10–19 years in that county.

Covariates were included in each of the models based on hypothesized links to stocking and delivery of meningococcal vaccines (see Sect. 1, Table S1 of the Supplemental Material).

All models accounted for spatial correlation in the outcomes through inclusion of county-level random effects that were modeled using a form of the conditional autoregressive (CAR) model. CAR models use neighbors of each county (i.e., those with touching borders) to describe spatial variability in the data and allows the data to determine how much of the total variability is spatially structured.

All models were fitted in a Bayesian framework using the *INLA* package in R. *INLA* provides an accurate approximation for marginal posterior inference and can be computationally efficient compared to a full Markov chain Monte Carlo sampling solution. All parameters were assigned weakly informative prior distributions (see Supplemental Material Sect. 2 for more information). Posterior means, standard deviations (SDs), and 95% credible intervals (CrIs) are used to represent point estimates and quantify uncertainty. Summary statistics of vaccine uptake are expressed as population-weighted medians and interquartile ranges, as calculated with the *ewcdf* and weighted quantile functions in the *spatstats* package in R.

## Results

**Fig. 3:**
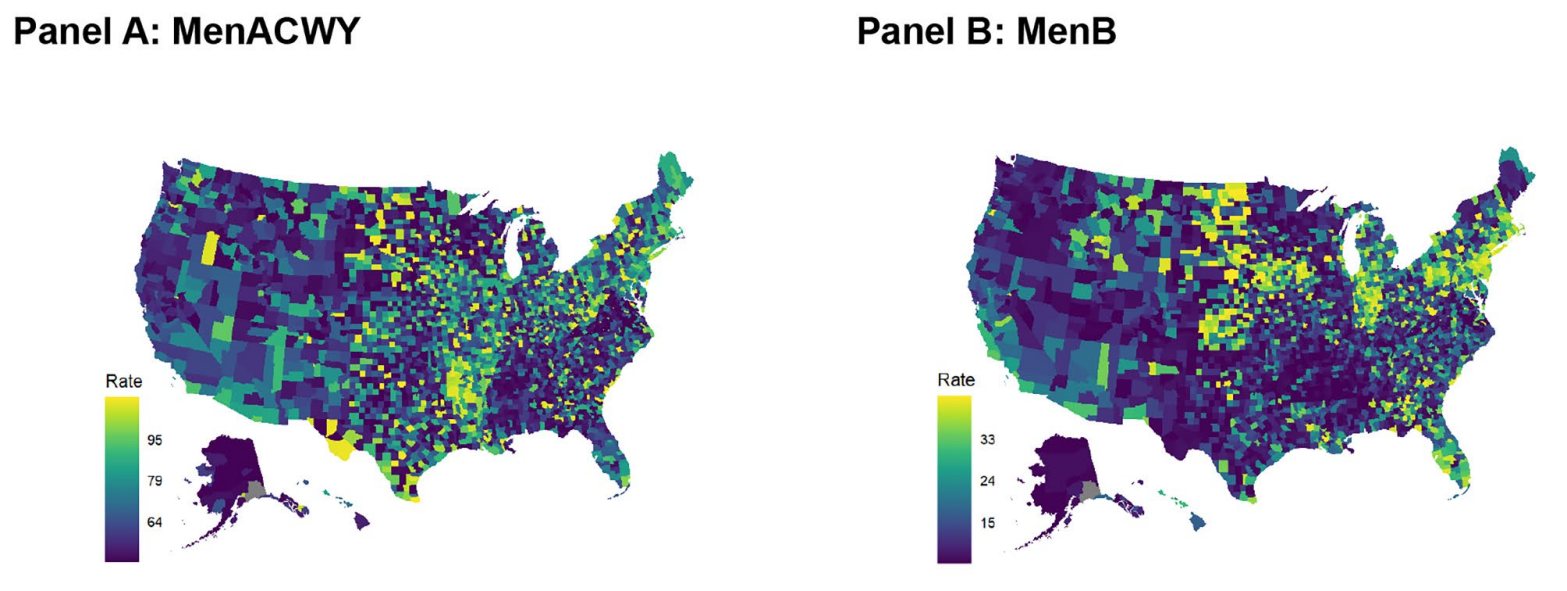
Fitted values of spatial regression model for total doses The scale of the two maps is different and they should not be directly compared. The dark colors indicate less stocking of vaccines per capita. The numbers on the scale show the population-weighted median and interquartile range

The stocking rates of MenACWY and MenB vaccines exhibit significant variability across counties (Fig. 3). Generally, MenACWY vaccines were more readily available in comparison to MenB across all counties in the US (Table 1). In the period from 2016 to 2019, there were a total of 81 doses of MenACWY distributed per 100 adolescents and 26 doses of MenB per 100 adolescents. There was substantial variability in rates of doses distributed by county, with a median of 62 doses/100 people (interquartile range: 42–80 doses/100) for MenACWY and 12 doses/100 people (interquartile range: 4–24 doses/100 people) for MenB. Population weighted means stratified by public and private market as well as SES are shown in Table 1. For both vaccines, overall fewer doses were available in areas with lower SES compared to areas with higher SES. However, the MenB stocking rate was more impacted (almost 30% reduction in counties with lower SES) by SES than MenACWY stocking (approximately 6% reduction in stocking) (Table 1).

**Table 1:** Availability of MenB and MenACWY in public and private markets*

	Socioeconomic Status	Total doses/100 adolescents	Public doses/100 adolescents	Private doses/100 adolescents
**MenB**	**Total**	**26**	**11**	**15**
	High	28	8	20
	Medium	26	12	14
	Low	20	13	7
**MenACWY**	**Total**	**81**	**40**	**41**
	High	80	32	48
	Medium	84	43	41
	Low	75	50	25

*Values are population-weighted means

Note: Low, Median and High SES is based on the first, second and third tertile of SVI, respectively

**Fig. 4:**
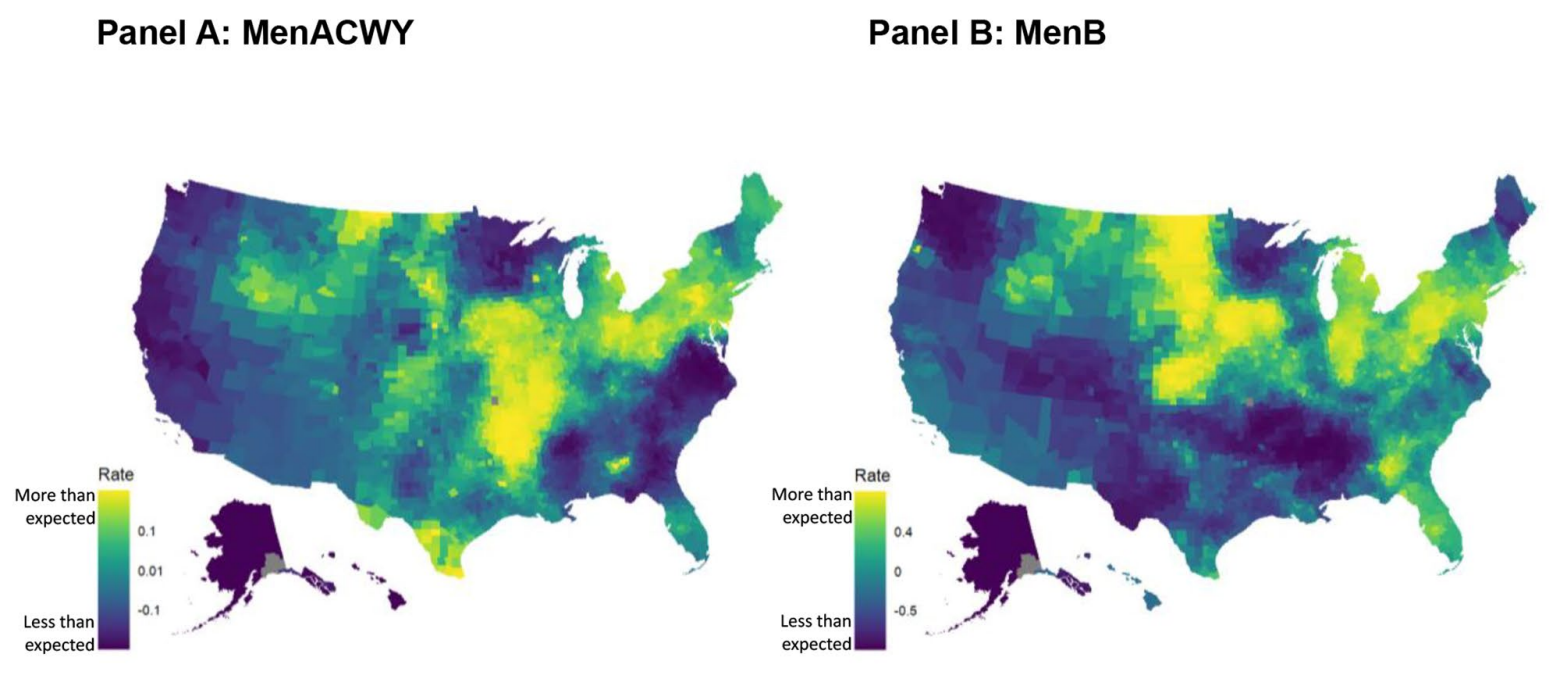
Unexplained variation in stocking rates between counties

The analysis of spatial variation in vaccine stocking (Table 2) showed that stocking of MenB was higher in counties with a higher number of pediatricians and primary care providers (PCP) per capita and in states with recommendations for MenB vaccination at age 16. For MenACWY, the main variables that were associated with stocking were again the number of pediatricians and PCPs per capita and the presence of a military training base. For each vaccine separately, SES was not a significant predictor of overall vaccine stocking when accounting for other factors such as universal purchase programs and vaccine school mandates. After accounting for potential confounding factors like socioeconomic factors and universal purchasing programs, a substantial unexplained spatial variability persisted in rates of stocking of MenB compared to MenACWY (Fig. 4).

**Table 2:** Key regression results for MenACWY and MenB doses per 1000 adolescents aged 10–19

Predictors	Stocking rate (Gross doses)				Public vs. private proportion of doses			
	MenACWY		MenB		MenACWY		MenB	
	Risk Ratio	95% CrIs	Risk Ratio	95% CrIs	Odds-Ratio	95% CrIs	Odds-Ratio	95% CrIs
SVI subscale, socioeconomic status​	1.00​	0.99, 1.02	0.97​	0.94, 1.01	1.18*​	1.14, 1.22	1.20*​	1.15, 1.25
State Universal Purchase Policy^†^	0.88​	0.77, 1.00	1.12​	0.84, 1.47	13.67*​	9.97, 18.21	13.20*​	9.30, 18.38
SVI subscale, socioeconomic status + Universal Purchasing Policy^†^	0.97​	0.94, 1.00	0.99​	0.93, 1.05	0.98​	0.90, 1.05	1.09​	0.99, 1.20
SVI subscale, socioeconomic status​ + State Recommendation for MenACWY/MenB, age 16​^††^	--	--	1.03	0.98, 1.09	--	--	1.12*	1.04, 1.21
SVI subscale, household composition and disability​	1.02*​	1.01, 1.03	1.02​	0.99, 1.04	1.01​	0.99, 1.04	1.01​	0.98, 1.04
SVI subscale, minority status and language​	1.03*​	1.02, 1.04	1.06*​	1.03, 1.08	1.00​	0.98, 1.03	1.02​	0.99, 1.05
SVI subscale, housing type & transportation​	1.03*​*​	1.02, 1.04	1.03*​	1.01, 1.05	0.96*​	0.94, 0.98	0.96*​	0.93, 0.98
State Requirement for MenACWY, age 11–12 ​	1.13​	1.00, 1.26	--	--	0.56*​	0.42, 0.71	--	--
State Recommendation for MenACWY, age 11–12 ​	0.84​	0.67, 1.05	--	--	0.17*​	0.09, 0.29	--	--
State Requirement for MenACWY, age 16​	1.16​	1.04, 1.29	--	--	1.31*​	1.03, 1.66	--	--
State Recommendation for MenACWY/MenB, age 16​^††^	1.18​	0.98, 1.41	1.48*​	1.22, 1.79	3.75*​	2.29, 5.76	1.24​	0.96, 1.56
Total pediatricians per 10,000 children (tertile 2)​	1.10*​	1.04, 1.16	1.38*​	1.23, 1.54	0.65*​	0.57, 0.73	0.64*​	0.55, 0.74
Total number of pediatricians per 10,000 children (tertile 3)​	1.28*​	1.20, 1.36	1.74*​	1.53, 1.97	0.66*​	0.57, 0.76	0.59*​	0.49, 0.69
Primary care providers per 10,000 residents (tertile 2)​	1.26*​	1.20, 1.36	1.28*​	1.15, 1.41	0.65*​	0.58, 0.72	0.67*​	0.58, 0.77
Primary care providers per 10,000 residents (tertile 3)​	1.56*​	1.47, 1.66	1.67*​	1.48, 1.89	0.45*​	0.39, 0.52	0.47*​	0.39, 0.55
Micropolitan Area	1.09*	1.04, 1.15	1.03	0.93, 1.14	0.90​	0.80, 1.01	0.94​	0.82, 1.07
Rural Area	1.19*​	1.11, 1.27	1.02​	0.88, 1.17	1.01​	0.85, 1.19	1.06​	0.87, 1.38
Basic training military base​	9.82*​	6.63, 14.09	--	--	0.14*​	0.06, 0.28	--	--
SVI subscale, socioeconomic status​ + State Recommendation for MenACWY/MenB, age 16(= 1)^††^	--	--	1.01	0.94, 1.07	--	--	1.35*	1.24, 1.47
SVI subscale, socioeconomic status​ + Universal Purchasing Policy for MenACWY/MenB(= 1)^†^	0.97	0.94, 1.01	0.96	0.90, 1.03	1.14*	1.06, 1.25	1.31*	1.18, 1.46
SVI subscale, socioeconomic status​ + State Recommendation for MenACWY/MenB(= 1)^††^, age 16 + Universal Purchasing Policy for MenACWY/MenB(= 1)^†^	--	--	0.99	0.91, 1.09	--	--	1.48*	1.30, 1.68

* Significant at a 5%-significance level

^†^For MenB public and private: Universal State Purchasing Policy for MenB. For MenACWY public and private: Universal State Purchasing Policy for MenACWY

^††^ For MenB public and private: State Recommendation for MenB. For MenACWY public and private: State Recommendation for MenACWY

When controlling for the presence of universal purchasing programs and school recommendations for vaccination, MenB stocking increased on average vs. MenACWY in counties with higher SES (see Tables 3 and 4). In counties without a universal purchasing program and without a recommendation for MenB at age 16, a 50% increase in SES (i.e., from 25 to 75%) was associated with a 35% increase of MenB stocking relative to MenACWY (Risk Ratio [RR]: 1.35, 95% Confidence Interval [CI]: 1.16, 1.57. Table 4). However, in counties with recommendation for MenB vaccination, the association between social vulnerability and differences in stocking is blunted (Table 4). State recommendations for MenACWY at age 11–12 and age 16 shifts the stocking of meningococcal vaccines towards increased stocking of MenACWY while the recommendations for MenB vaccines shift the vaccine stocking towards relatively more MenB doses. The results further emphasize the relatively higher importance of state recommendations for MenACWY at age 11–12 than at age 16 (Table 3). Moreover, the results show that counties that were more rural and had fewer PCPs per capita tended to have lower rates of stocking of MenB relative to MenACWY (Table 3). The presence of a military base also played a role in enhancing the stocking gap.

**Table 3:** Key regression results for MenACWY to total gross meningococcal vaccine doses

Predictor​	Risk-Ratio​	95% CI​
SVI subscale, socioeconomic status​	1.06*	1.03, 1.09
SVI subscale, minority status and language​	0.96*	0.94, 0.98
State Recommendation for MenACWY, age 11–12 ​	3.05*	1.52, 5.52
State Requirement for MenACWY, age 16​	1.34*	1.00, 1.77
State Recommendation for MenB, age 16​	0.39*	0.28, 0.53
Total pediatricians per 10,000 children (tertile 2 vs. tertile 1)​	0.72*	0.65, 0.79
Total number of pediatricians per 10,000 children (tertile 3 vs. tertile 1)​	0.67*	0.60, 0.75
Primary care providers per 10,000 residents (tertile 3 vs. tertile 1)​	0.85*	0.77, 0.95
Rural Area	1.29*	1.12, 1.48
Basic training military base (ref: no military base)​	2.48*	1.32, 4.22

* Significant on a 5%-significance level

Note: Non-significant variables not shown

**Table 4:** Association between county-level socioeconomic measurement and relative stocking of MenB vs. MenACWY

	County-level socioeconomic measurement	School recommendation for MenB vaccine at age 16		No MenB vaccine recommendation at age 16	
		OR	95% CI	OR	95% CI
Universal purchasing state - MenACWY	50% increase in SVI score	1.16	0.73, 1.77	1.42	0.98, 2.00
No universal purchasing for MenACWY	50% increase in SVI score	1.10	0.81, 1.47	1.35	1.16, 1.57

Key: CI – confidence interval; OR – odds ratio; SVI – social vulnerability index

Note: The table shows variations by school recommendations for MenB and MenACWY universal purchasing policies

While overall nearly the same amount of MenACWY doses were available on the public and private market, on average the number of MenB doses on the public market was lower by one third compared to the private market. Private doses were mainly available in counties with high SES while the stocking of public doses was two times as large as the private stock in areas with low SES (Table 1).

The results of the spatial regression analysis comparing the availability of public vs. private doses (Table 2) showed that lower SES was associated with a shift towards stocking doses on the public vs. private market. Having universal purchasing in the state was associated with a strong, nearly complete, shift towards public stocking of both vaccines. There was also an association between a higher proportion of public stocking and vaccination requirements at age 16 for MenACWY while the recommendation at age 11 was associated with a shift towards private doses. The relationship was not significant for MenB vaccines. Finally, other factors associated with a shift towards public doses were the presence of a tribal health care facility, a higher proportion of the population on Medicaid, and a higher proportion of uninsured individuals. Having more pediatricians was associated with more doses on the private market. For MenACWY, the proportion of public vs. private doses was significantly lower in counties with military training bases.

## Discussion

This study sought to understand the complexities of disparities in vaccine availability, measured as stocking of vaccine doses, for MenACWY and MenB vaccines across the US, focusing on the role of SES, universal purchase programs, and school vaccination recommendations. To our knowledge, it is the first study of its kind to use spatial regression to investigate how socioeconomic factors and state level policies are associated with differences in vaccine stocking at the county level in the US describing inequalities in stocking of meningococcal vaccination.

Stocking can be considered a proxy for access, given the amount of asymmetric information present in this sector [14]. A US study analyzing stocking variations across vaccines for adults found that, with the exception of influenza vaccination, there are large variations in stocking across vaccines and organizations [23]. Interestingly the study found that the main reasons for not stocking a vaccine were “not a priority for our practice/organization” (varying from 54 to 80% among respondents) and the cost of purchasing and maintaining the vaccine stock. Moreover, the study found that larger practices (6 or more physicians) were more likely to stock all vaccines than smaller practices. These results are particularly interesting given that under the Affordable Care Act, all vaccines incorporated into the ACIP’s immunization schedules (including SCDM recommendations) are covered without imposing any form of cost-sharing, such as co-payments, co-insurance, or deductibles to patients.

Our study unveiled disparities in stocking between MenACWY and MenB vaccines. The stocking of MenACWY vaccines is approximately three times higher than MenB vaccines across the US, with large heterogeneity across states and counties, SES, and in both public and private markets. Even after controlling for observable factors that likely influence the stocking of meningococcal vaccines at regional level, such as state vaccine policies and access to health providers, significant unexplained variation in stocking remains. One unobservable factor that could influence stocking decisions of providers is the type of recommendation, i.e., age-based routine recommendation (for MenACWY) and SCDM (for MenB). The perceived opportunity cost of purchasing and maintaining a vaccine with a SCDM recommendation may be considered higher for MenB than MenACWY. Moreover, evidence suggests that SCDM is not fully understood by primary care providers in terms of coverage implications, and it poses challenges in communicating the recommendations to patients [24].

At the same time, parents, on average, tend to rely heavily on physician recommendations for vaccines. For instance, a recent study by Coulter et al. (2024), analyzed qualitatively the major determinants of meningococcal vaccination preferences among children and their families. The results highlighted that some parents struggle to remember which vaccines (MenACWY and MenB) their children had received and that some would rely on their physician to keep track of the vaccines their children received [25, 26]. This combination of parental unawareness and gaps in physician knowledge underscores substantial obstacles in the case of SCDM recommendations and highlights the importance of further education for physicians, as parents are less likely to raise the topic themselves due to their limited awareness [25].

In a recent meeting, the ACIP has discussed to alter the MenB recommendation to either a risk-based (i.e., college students) or an age-based routine recommendation, prompted by several factors including poor uptake, missed opportunities, the absence of a strong recommendation, and challenges faced by clinicians in comprehending the recommendation [27]. This consideration stems from research indicating that college students face a significantly higher risk of serogroup B disease compared to non-college students. Studies have shown that college students have a 3.5-fold (95% CI: 2.2–5.4) higher risk of serogroup B disease, with incidence peaking for 19-year-old college students and declining after age 20. On-campus residents were found to have a 2.9-fold (95% CI: 1.8–4.6) higher risk of serogroup B disease compared to off-campus residents, and students participating in Greek life were at a 9.8-fold (95% CI: 4.6–21.2) higher risk of serogroup B disease during outbreaks compared to other students [27]. These findings highlight the importance of targeted vaccination strategies for college students to mitigate the risk of serogroup B disease outbreaks.

Our study highlights the effectiveness of school mandates and recommendations as a strategy to enhance stocking rates and reduce stocking variability across both MenB and Men ACWY vaccines, thus improving access to meningococcal vaccines. In particular, the presence of school recommendations for MenB was associated with narrowing the gap between MenB and MenACWY stocking across SES levels. Our findings are consistent with the literature, which suggests that school-mandated vaccinations lead to higher levels of uptake within the population and are one of the most efficacious strategies for implementation in adolescents [28–30], suggesting that “mandates, when initiated with care, are one of the most effective implementation strategies for adolescents” [31].

The results of our study show that socioeconomic disparities were particularly important in counties with no school recommendation for MenB vaccination, with counties with higher SVI reporting a lower gap in stocking of MenB vs. MenACWY vaccines. Vaccines are fully reimbursed for children independently of insurance status. The presence of universal purchasing programs does not impact the stocking of doses per se, however the number of public vs. private doses tend to be higher in the presence of such programs.

Counties that were more rural and had fewer PCPs per capita tended to have lower stocking of MenB vaccines relative to MenACWY, whereas counties with more pediatricians and PCPs had significantly more doses of both vaccines. These findings suggest an overall inequitable distribution of meningococcal vaccines across the US and providers, particularly impacting access to MenB vaccination.

Previous studies that tried to investigate the relationship between uptake of MenACWY and MenB vaccine and individual and regional SES [11, 20, 32] showed ambiguous links with income levels for MenACWY while for MenB, the link for SES seems to be clearer [27, 28]. For instance, Niccolai et al. (2019) found mixed outcomes in MenACWY doses related to income levels, while Pruitt et al. (2022) pointed to variations in the uptake of the first dose [20, 28]. Among individuals with lower SES, Pingali et al. (2023) found comparable rates for the first dose but decreased rates for the second dose [11]. As for MenB, separate research underscores a greater adoption of the vaccine in more affluent neighborhoods [27] while contrasting findings propose no noteworthy divergence in MenB utilization linked to poverty levels [33]. These combined findings highlight the intricate interplay between vaccine usage and socioeconomic factors in a complex health care system like the US, underscoring the need for more in-depth research to gain a comprehensive understanding and address these differences. Our study adds to this literature by analyzing the relationship between regional SES and stocking of meningococcal vaccines as a proxy for access to vaccines and thus highlighting regional access barriers associated to inequality as well as leveraging a variety of data sources and advanced methodological tools to evaluate the inequality.

### Strengths and limitations

This study describes the large variability in meningococcal vaccination access across states and counties, and the role that SES, public and private purchase programs, and differential recommendations play in determining vaccine stocking patterns. The models were able to explain much of the variability although unexplained spatial variability persisted. This study uses very diverse datasets, offers a unique perspective on vaccine stocking rates, as well as using spatial regression models to model the availability of meningococcal vaccines on county level in the US. This provided the opportunity to investigate a variety of factors that may be associated with vaccine stocking, considering the influence of neighboring counties. We believe this research significantly adds to our knowledge of the factors influencing vaccine access and opens the door to more innovative studies to try to understand the key drivers of inequalities and access.

The study results may imply that SCDM is also a driver of differences in access to and uptake of MenB vaccines. However, it is not possible to measure SCDM and therefore the influence of SCDM on the differences in stocking of MenACWY vs. MenB vaccines could not directly be analyzed in this study.

Moreover, the study does not analyze the impact of migration on stocking on both vaccines due to the lack of data sources. The inclusion of college recommendations for meningitis vaccination may be another interesting area of research, analyzing how movement between states during college may influence vaccination stocking. Unfortunately, data were not available to address these questions.

Further limitations arise from the unavailability of data, as only estimates of the total number of eligible children in each county were available, which precluded stratification into the number of children eligible for publicly funded doses. The analyses that compare relative stocking of MenACWY and MenB partially addresses this by assuming the eligible population in the county for MenB and MenACWY for public or private markets is the same and allows for analysis of the public and private markets separately. Also, the availability for the stocking data (2016 to 2019) is slightly different from the available Census Bureau data that was used for other variables including the estimation of SVI (2015 to 2019).

Furthermore, the stocking data has two limitations. First, the IQVIA data used in this analysis represent stocking and not administration of the vaccines. They served as variables for the vaccines’ availability and as a proxy for access to meningococcal vaccines and not as a measure of uptake. Second, the stocking data accounts for all doses and not only those doses intended for adolescent vaccination, which might lead to an overestimation on the private market since risk-based vaccinations of other population groups including infants and adults are not considered. However, given the generally low uptake of vaccines in risk groups, the impact is expected to be small [34]. Lastly, it should be noted that, while widely used in public health, the CDC’s SVI was initially developed to understand natural disaster risk on populations. In our study we make secondary use of the data.

## Conclusions

Our study pioneers a spatial regression approach for assessing vaccine accessibility, yielding valuable insights into the impact of socioeconomic disparities and other state-level policies at the county level in the US. Disparities in the stocking of MenACWY and MenB vaccines are present across the US, with more pronounced barriers for MenB. Nationally, the stocking of MenACWY is approximately three times higher than the stocking of MenB, yet this discrepancy varies across counties, SES, and market type. Counties with lower SES had reduced vaccine availability of MenB compared to MenACWY, with public sources being the primary provider for both vaccines in these areas. Similarly, rural areas with fewer pediatricians and PCPs had fewer doses of MenB, while counties with a greater number tended of pediatricians and PCPs tend to have more vaccines. Finally, the study shows the importance of school recommendations to narrow the SES gap in general and particularly between MenB and MenACWY stocking. Although SCDM could not be directly assessed as a determinant of variability in stocking rate at the national, state, and local level, the results of our study may refer to the importance that a differential recommendation has on the information and knowledge of physicians, parents, educators, and patients between vaccines, and therefore, on demand and stocking.

Our findings highlight the need for comprehensive strategies to establish equitable vaccine distribution. Promoting meningococcal vaccination in adolescents and young adults is an important health policy objective and reducing access barriers for vaccination remains a key target. School recommendations at the state level could positively increase the availability of vaccines by increasing awareness among health care providers, parents, and schools about the importance of vaccination against meningococcal disease and may contribute to decreasing inequalities in particular, in lower SES areas. In particular, the access to meningococcal stocking should not depend on the number of pediatricians or SES within the area of residence. Access should be equitable and vaccine uptake should be based on risk-based, independent of the type of recommendation (SCDM vs. full recommendation). Incorporating equity considerations into the ACIP EtR is a first step to mitigating disparities during guideline development. Looking ahead, there is a pressing need for coordinated efforts towards ensuring equitable vaccine recommendations. Such measures are necessary for maintaining the health of all individuals, regardless of their socioeconomic background or geographic location.

### Electronic supplementary material

Below is the link to the electronic supplementary material.

Supplementary Material 1

## Data Availability

The data that support the findings of this study are available from IQVIA but restrictions apply to the availability of these data, which were used under license for the current study, and so are not publicly available. Data are however available from the authors upon reasonable request and with permission of IQVIA.
