# Supplementary material for: The impact of regional disparities on the availability of meningococcal vaccines in the US
Source: BMC Public Health. 2024 Jul 3;24:1771. doi: 10.1186/s12889-024-19081-w (PMC11221024; doi:10.1186/s12889-024-19081-w)
Supplement: Supplementary file 1 — Supplementary Material 1 [file 12889_2024_19081_MOESM1_ESM.docx]

**Supplementary Material**

Section 1. Methodology - Variable descriptives and sources

**Table S1.** Variable description

| **Variable** | **Role** | **Data source(s)** | **Operational definition** |
| --- | --- | --- | --- |
| MenACWY Stocking per Capita (Public) | Outcome | CDC VFC data and the ACS 2015-2019 5-year estimates for 10-19 year-old residents | Cumulative, gross deliveries of MenACWY vaccine doses via the VFC program per 1,000 adolescents aged 10-19 between 2016 and 2019; counties with no deliveries are assigned a 0 |
| MenACWY Stocking per Capita (Private) | Outcome | IQVIA DDMD and the ACS 2015-2019 5-year estimates for 10-19 year-old residents | Cumulative, gross deliveries of MenACWY vaccine doses via traditional wholesale/sales channels per 1,000 adolescents aged 10-19 between 2016 and 2019; counties with no deliveries are assigned a 0 |
| MenACWY Stocking per Capita (Total) | Outcome | IQVIA DDMD, CDC VFC data, and the ACS 2015-2019 5-year estimates for 10-19 year-old residents | Total cumulative, gross doses of MenACWY vaccine via both public and private channels per 1,000 adolescents aged 10-19 between 2016 and 2019; counties with no deliveries are assigned a 0 |
| MenB Stocking per Capita (Public) | Outcome | CDC VFC data and the ACS 2015-2019 5-year estimates for 10-19 year-old residents | Cumulative, gross deliveries of MenB vaccine doses via the VFC program per 1,000 adolescents aged 10-19 between 2016 and 2019; counties with no deliveries are assigned a 0 |
| MenB Stocking per Capita (Private) | Outcome | IQVIA DDMD and the ACS 2015-2019 5-year estimates for 10-19 year-old residents | Cumulative, gross deliveries of MenB vaccine doses via traditional wholesale/sales channels per 1,000 adolescents aged 10-19 between 2016 and 2019; counties with no deliveries are assigned a 0 |
| MenB Stocking per Capita (Total) | Outcome | IQVIA DDMD, CDC VFC data, and the ACS 2015-2019 5-year estimates for 10-19 year-old residents | Total cumulative, gross doses of MenB vaccine via both public and private channels per 1,000 adolescents aged 10-19 between 2016 and 2019; counties with no deliveries are assigned a 0 |
| SVI | Exposure | ACS 2015-2019 5-year estimates | Social vulnerability index, overall percentile score between 0 and 1 |
| SVI, SES Subscale | Exposure | ACS 2015-2019 5-year estimates | Social vulnerability index, socioeconomic subscale percentile score between 0 and 1 |
| SVI, HCD Subscale | Exposure | ACS 2015-2019 5-year estimates | Social vulnerability index, household composition & disability subscale percentile score between 0 and 1 |
| SVI, MSL Subscale | Exposure | ACS 2015-2019 5-year estimates | Social vulnerability index, minority status & language subscale percentile score between 0 and 1 |
| SVI, HTT Subscale | Exposure | ACS 2015-2019 5-year estimates | Social vulnerability index, housing type & transportation subscale percentile score between 0 and 1 |
| Pediatricians per Capita | Potential Confounder | American Board of Pediatrics Workforce Survey (currently 2021) and the ACS 2015-2019 5-year estimates | Number of providers certified in pediatrics without a second certification per 1,000 children aged 19 years or younger; counties without pediatricians are assigned a 0 |
| Primary Care Providers per Capita | Potential Confounder | County Health Rankings Data, 2020 Release and the ACS 2015-2019 5-year estimates | Number of primary care providers per 1,000 residents in 2019; counties without primary care providers are assigned a 0 |
| Percent of Children Receiving Public Health Insurance (Medicaid or CHIP) | Potential Confounder | ACS 2015-2019 5-year estimates | Number of children aged 18 or younger with Medicaid coverage per 1,000 children aged 18 or younger; implied reference category is all children with health insurance other than Medicaid/CHIP |
| Percent of Children without Health Insurance | Potential Confounder | ACS 2015-2019 5-year estimates | Number of children aged 18 or younger with no health insurance coverage per 1,000 children aged 18 or younger; implied reference category is all children with health insurance other than Medicaid/CHIP |
| Rural County | Potential Confounder | USDA’s Urban Influence Codes for Counties, 2013 | FALSE or TRUE; if TRUE, county has a UIC score of 9 or greater; reference category is “metropolitan county” |
| Micropolitan County | Potential Confounder | USDA’s Urban Influence Codes for Counties, 2013 | FALSE or TRUE; if TRUE, county has a UIC score between 3 and 8 ; reference category is “metropolitan county” |
| Metropolitan County | Potential Confounder | USDA’s Urban Influence Codes for Counties, 2013 | FALSE or TRUE; if TRUE, county has a UIC score of 1 or 2; this is the reference category |
| DoD Basic Training Program (regardless of health care facilities) | Potential Confounder | List of facilities (and associated counties) generated in consultation with Pfizer experts | FALSE or TRUE; if TRUE, county has an unclassified, active duty military base where basic training occurs, regardless of the level of health care available; reference category is “No DoD Installation” |
| DoD Major Medical Facility (hospital or medical center) | Potential Confounder | List of facilities and installations (and associated with counties) generated from Department of Defense data and New York Times reporting | FALSE or TRUE; if TRUE, county has an unclassified, active duty military base with a medical center or hospital, but no basic training program; reference category is “No DoD Installation” |
| DpD Small Medical Facility (clinic) | Potential Confounder | List of facilities and installations (and associated with counties) generated from Department of Defense data and New York Times reporting | FALSE or TRUE; if TRUE, county has an active duty military base with a clinic, but no basic training program and no hospital or medical center; reference category is “No DoD Installation” |
| DoD No Medical Facility | Potential Confounder | List of facilities and installations (and associated with counties) generated from Department of Defense data and New York Times reporting | FALSE or TRUE; if TRUE, county has an active duty military base but no military health care facility or basic training program; reference category is “No DoD Installation” |
| DoD Installation, No Active Duty | Potential Confounder | List of facilities and installations (and associated with counties) generated from Department of Defense data and New York Times reporting | FALSE or TRUE; if TRUE, county has an unclassified military base but it is either a National Guard or Reserve facility only; reference category is “No DoD Installation” |
| No DoD Installation | Potential Confounder | List of facilities and installations (and associated with counties) generated from Department of Defense data and New York Times reporting | FALSE or TRUE; if TRUE, county has no unclassified military installation; this is the reference category |
| Tribal Lands with Health Care Facility | Potential Confounder | List of Indian Health Service facilities and tribal lands (and associated with counties) generated from IHS and Census Bureau Data | FALSE or TRUE; if TRUE, county has tribal lands and at least one HIS facility; reference category is “No Tribal Lands” |
| Tribal Lands without Health Facility | Potential Confounder | List of Indian Health Service facilities and tribal lands (and associated with counties) generated from IHS and Census Bureau Data | FALSE or TRUE; if TRUE, county has tribal lands but no IHS facility; reference category is “No Tribal Lands” |
| No Tribal Lands | Potential Confounder | List of Indian Health Service facilities and tribal lands (and associated with counties) generated from IHS and Census Bureau Data | FALSE or TRUE; if TRUE, county has no tribal lands; this is the reference category |
| State Requirement | Effect modifier | Compiled by the study team |  |
| MenACWY, Required at 11-12 | Potential Confounder | Pfizer review of State Public Health Websites | FALSE or TRUE; if TRUE, state requires MenACWY at age 11 or 12 |
| MenACWY, Required at 16 | Potential Confounder | Pfizer review of State Public Health Websites | FALSE or TRUE; if TRUE, state requires MenACWY at age 16 |
| MenB, Recommended | Potential Confounder | Pfizer review of State Public Health Websites | FALSE or TRUE; if TRUE, state recommends MenB at age 16 |
| MenACWY, Universal Purchase | Potential Confounder | Pfizer Subject Matter Experts | FALSE or TRUE; if TRUE, state has a universal purchase policy for MenACWY vaccines |
| MenB, Universal Purchase | Potential Confounder | Pfizer Subject Matter Experts | FALSE or TRUE; if TRUE, state has a universal purchase policy for MenB vaccines |

**Vaccine for Children (VFC)**

Under the Affordable Care Act, also known as Obamacare, enacted in 2010, ACIP recommendations that have been incorporated in the CDC’s immunizations schedules are generally required to be reimbursed by health insurers without introducing any type of cost-sharing scheme, such as a co-payment, co-insurance, or deductible. This applies to SCDM recommendations as well if they are listed in the CDC immunization schedule [1][2].

Likewise, infant, young children and adolescent vaccines recommended by the ACIP are covered by the Vaccine for Children (VFC) program. This allows minors to get vaccinated even if their parents cannot afford vaccinations. The funding for this program is channeled from the Centers for Medicare and Medicaid Services to the CDC. In turn, the CDC buys the vaccines for a discounted price and allocates them to grantees, or state health departments and local and territorial public health agencies. These redistribute the vaccines to VFC providers at no cost. These can be both public health clinics and private physician’s offices [1][2]. Approximately 50% of children aged <19 years old receive their vaccines through the VFC program [3].

**Public Stocking Data**

There are several criteria a minor must meet to be eligible for the VFC program. He or she must be younger than 19 years old and either be Medicaid-eligible, uninsured, underinsured, or American Indian/Alaska Native. Vaccines provided by a VFC provider to eligible children are completely free. However, there are other charges associated with a vaccination that are not reimbursed. For example, doctors can set an administration fee for each vaccine. However, parents of VFC eligible children who cannot afford this surcharge will not be obligated to pay for it [1][2].

The VFC data contained 5-digit FIPS codes for the county where the delivery took place as well as a month-date combination. The DDMD data were delivered alongside the Pfizer Best Address table data, which includes street addresses and USPS 5-digit ZIP Codes for outlets that may receive shipments of medications and/or vaccines. Using the address and the ZIP Codes, outlets were “geocoded” using the following workflow:

1. If an outlet had a street address in the Pfizer Best Address table, this was passed to the US Census Bureau’s batch geocoding API using the R package *censusxy.*
   1. If an outlet’s address resulted in an API match, the approximate latitude and longitude of the address was than compared to a national data set of county boundaries to locate the county that the address was located within.
2. If an outlet’s address did not result in an API match, or if it did not have an address, its 5-digit USPS ZIP Code was then used in combination with the US Department of Housing and Urban Development’s ZIP to County crosswalk files to identify the correct 5-digit FIPS code for outlets.
3. For the small number of remaining outlets, the USPS ZIP Code was converted to a US Census Bureau ZIP Code Tabulation Area (ZCTA), whose geographic midpoint (“centroid”) was than compared to a national data set of county boundaries to locate the county that the address was located within.

Once all the deliveries were paired with a 5-digit FIPS code for individual counties, the deliveries were summarized using the county FIPS code and the delivery year to create counts per county for the 2016 to 2019 study period. The US Census Bureau’s population estimate for 10- to 19-year-olds from the 2015 to 2019 5-year ACS to create gross delivery rates per 1,000 children in that age cohort.

**Social Vulnerability Index**

**Table S2.** Variables and Themes Included in the Social Vulnerability Index Databases [4]

| **Overall Vulnerability** | **Socioeconomic status** | Below poverty |
| --- | --- | --- |
|  |  | Unemployed |
|  |  | Income |
|  |  | No High School Diploma |
|  | **Household composition and disability** | Age 65 or Older |
|  |  | Age 17 or Younger |
|  |  | Older Than Age 5 With a Disability |
|  |  | Single-Parent Households |
|  | **Minority status and language** | Minority |
|  |  | Speaks English “Less Than Well” |
|  | **Housing and transportation** | Multiunit Structures |
|  |  | Mobile Homes |
|  |  | Crowding |
|  |  | No Vehicle |
|  |  | Group Quarters |

In our analysis, our script was validated by successfully replicating the CDC’s 2018 SVI scores. The CDC warns that a complete replication of their work is impossible due to rounding error introduced in their workflow when percentiles are calculated. Our validation work verified that the source data were identical, and that the calculated scores were close or identical to the CDC’s data. SVI scores were rounded to four decimal places to match the CDC’s method of reporting. The mean difference between our scores and the CDC’s was -0.00001, with a standard deviation of 0.00443.

**State Requirements**

**Table S3.** State requirements

| **State** | **MenACWY (Ages 11-12)** | **MenACWY (Age 16)** | **MenB (Age 16)** |
| --- | --- | --- | --- |
| **Alabama [5]** | Recommended | Recommended | Recommended |
| **Alaska [6]** | No | No | No |
| **Arizona [7]** | Required | No | No |
| **Arkansas [8]** | Required | Required | No |
| **California [9]** | No | No | No |
| **Colorado [10]** | No | No | No |
| **Connecticut [11]** | Required | No | No |
| **Delaware [12]** | Required | No | No |
| **Florida [13]** | No | No | No |
| **Georgia [14]** | Required | Required | No |
| **Hawaii [15]** | Required | Required | No |
| **Idaho [16]** | No | No | No |
| **Illinois [17]** | Required | Required | No |
| **Indiana [18]** | Required | Required | Recommended |
| **Iowa [19]** | Required | Required | No |
| **Kansas [20]** | Required | Required | No |
| **Kentucky [21]** | Required | Required | No |
| **Louisiana [22]** | Required | Required | Recommended |
| **Maine [23]** | Required | Required | No |
| **Maryland [24]** | Required | Required | Recommended |
| **Massachusetts [25]** | Required | Required | No |
| **Michigan [26]** | Required | No | No |
| **Minnesota [27]** | Required | Required | No |
| **Mississippi [28]** | No | No | No |
| **Missouri [29]** | Required | Required | No |
| **Montana [30]** | No | No | No |
| **Nebraska [31]** | No | No | No |
| **Nevada [32]** | Required | Required | No |
| **New Hampshire [33]** | No | No | No |
| **New Jersey [34]** | Required | No | No |
| **New Mexico [35]** | Required | Recommended | No |
| **New York [36]** | Required | Required | No |
| **North Carolina [37]** | Required | Required | No |
| **North Dakota [38]** | Required | Required | No |
| **Ohio [39]** | Required | Required | No |
| **Oklahoma [40]** | Recommended | Recommended | Recommended |
| **Oregon [41]** | No | No | No |
| **Pennsylvania [42]** | Required | Required | No |
| **Rhode Island [43]** | Required | Required | No |
| **South Carolina [44]** | No | No | No |
| **South Dakota [45]** | Required | Recommended | No |
| **Tennessee [46]** | No | No | No |
| **Texas [47]** | Required | No | No |
| **Utah [48]** | Required | No | No |
| **Vermont [49]** | Required | Required | No |
| **Virginia [50]** | Required | Required | No |
| **Washington [51]** | Required | Required | No |
| **West Virginia [52]** | Required | Required | No |
| **Wisconsin [53]** | No | No | No |
| **Wyoming [54]** | Recommended | Recommended | Recommended |
| No = Not mentioned on the website OR recommended only for those at risk  Required = Required for entry  Recommended = “Recommended” or “should” | | | |

**Department of Defense (DoD) Medical Facilities and Basic Training Sites**

DoD installation locations were obtained from data.gov. The clinic locations were matched with installations by geographic proximity using a geographic information system (GIS) technique called a ‘spatial join’. All matches were manually verified based on facility names. The DoD data themselves did not consistently separate hospitals and medical centers from clinics. To separate major medical facilities from clinics, data from a 2014 *New York Times* story on DoD hospitals were paired with the matched installation and health care facilities data [55]. The resulting combined health care facilities data set was manually verified to ensure that each of the 117 facilities was correctly labeled as a clinic, hospital, or medical center. Each facility’s capabilities were verified with publicly available data on the TRICARE website. All manual data entry with corrections from the TRICARE website were quality checked by a second researcher for accuracy.

Once quality checks were complete, bases were labeled with the highest level of on-base care (since a few bases contain multiple facilities; one of none, clinic, hospital, medical center) and at the counties level using a GIS spatial join technique. Given the complexity of the data reporting, different labels were created. A final, single measure of DoD presence was developed with the following levels:

- No unclassified installation
- Unclassified installation for national guard or reserve components only
- Unclassified, active-duty installation but no health care facility and no basic training program
- Unclassified, active-duty installation with a clinic and no basic training program
- Unclassified, active-duty installation with a hospital or medical center and no basic training program
- Unclassified, active-duty installation with a basic training program regardless of health care facility status

**Number of Health care Providers**

Counts of health care providers were sourced from two databases. The number of pediatricians was sourced from the American Board of Pediatrics 2020 to 2021 Workforce Survey [56], and the overall number of primary care providers was sourced from the 2020 County Health Rankings project estimates, which use 2019 National Provider Identification data from CMS. In both cases, ACS data were used for a denominator to calculate per capita rates of health care providers.

**Rurality**

The USDA’s Urban Influence Codes for Counties data set is the source of measures related to how urban or rural a county is and was selected following guidance in Long et al. 2021 [57].

**Tribal Lands and Indian Health Service Facilities**

Our measures of indigenous lands and health care access utilized tribal land boundary data from the Census Bureau and health care facility location data from the Indian Health Service (IHS). We overlapped both with US county boundaries to create logical indicators for the presence of tribal lands in a county as well as the presence of several different types of health facilities – IHS facilities, federally funded indigenous urban health clinics, and tribal clinics. We then summarized these data in a single variable capturing a county’s status as having no tribal lands or health care, only tribal lands, non-IHS health care, or IHS health care facilities.

Since some counties contain multiple installations, data were aggregated into 2 measures:

(1) The highest level of care available within the county, 1 of the following:

- No unclassified installation
- Unclassified installation for national guard or reserve components only
- Unclassified, active-duty installation but no health care facility
- Unclassified, active-duty installation with a clinic
- Unclassified, active-duty installation with a hospital
- Unclassified, active-duty installation with a medical center

(2) A measure of whether any facility within the county houses a basic training program (TRUE or FALSE). For installations that crossed county boundaries, every enclosing county received the same values regardless of the geographic location of the health care facility or basic training program.

Section 2. Modeling - Bayesian spatial regression models

**Negative Binomial Regression Modeling**

Likelihood:

$$Y_{i}|r,p_{i}\sim\mathrm{Negative} \mathrm{Binomial}\left( r,p_{i} \right), i=1,\ldots,n;$$

$$p_{i}=\frac{r}{\lambda_{i}+r}, \ln\left( \lambda_{i} \right)=O_{i}+\mathbf{x}_{i}^{T}\boldsymbol{\beta}+\phi_{i}$$

- $Y_{i}$: Outcome variable; observed count in county *i*
- $O_{i}$: Offset term; log of relevant population total in county *i*
- $\mathbf{x}_{i}$: Vector of county *i*-specific covariates

Spatial Process:

$$\phi_{i}|\boldsymbol{\phi}_{-i},\rho,\tau^{2}\sim N\left( \frac{\rho\sum_{j=1}^{n} w_{ij}\phi_{i}}{\rho\sum_{j=1}^{n} w_{ij}+1-\rho},\frac{\tau^{2}}{\rho\sum_{j=1}^{n} w_{ij}+1-\rho} \right)$$

- $w_{ij}$: Binary variable equal to one if counties *i* and *j* are neighbors, zero otherwise; $w_{ii}\equiv0$ by definition

Prior Distributions:

- $\ln\left( r \right)\sim N\left( {0,100}^{2} \right)$
- $\beta_{j}\sim N\left( {0,100}^{2} \right), j=1,\ldots,p_{x}$
- $\ln\left( \frac{\rho}{1-\rho} \right)\sim N\left( {0,100}^{2} \right)$
- $\tau^{2}\sim Inverse Gamma(0.01, 0.01)$

Model Fitting:

Approximate Bayesian marginal posterior inference using the Integrated Nested Laplace Approximation (INLA).

**Binomial Logistic Regression Modeling**

Likelihood:

$$Y_{i}|p_{i}\sim\mathrm{Binomial}\left( n_{i},p_{i} \right), i=1,\ldots,n;$$

$$\ln\left( \frac{p_{i}}{1-p_{i}} \right)=\mathbf{x}_{i}^{T}\boldsymbol{\beta}+\phi_{i}$$

- $Y_{i}$: Outcome variable; observed count in county *i*
- $n_{i}$: Denominator term in county *i*
- $\mathbf{x}_{i}$: Vector of county *i*-specific covariates

Spatial Process:

$$\phi_{i}|\boldsymbol{\phi}_{-i},\rho,\tau^{2}\sim N\left( \frac{\rho\sum_{j=1}^{n} w_{ij}\phi_{i}}{\rho\sum_{j=1}^{n} w_{ij}+1-\rho},\frac{\tau^{2}}{\rho\sum_{j=1}^{n} w_{ij}+1-\rho} \right)$$

- $w_{ij}$: Binary variable equal to one if counties *i* and *j* are neighbors, zero otherwise; $w_{ii}\equiv0$ by definition

Prior Distributions:

- $\beta_{j}\sim N\left( {0,100}^{2} \right), j=1,\ldots,p_{x}$
- $\ln\left( \frac{\rho}{1-\rho} \right)\sim N\left( {0,100}^{2} \right)$
- $\tau^{2}\sim Inverse Gamma(0.01, 0.01)$

Model Fitting:

Approximate Bayesian marginal posterior inference using the Integrated Nested Laplace Approximation (INLA).

Section 3. Results

**Regression Results**

**Figure S1.** Fitted values of spatial regression model for total doses

| Panel A: MenACWY | Panel B: MenB |
| --- | --- |


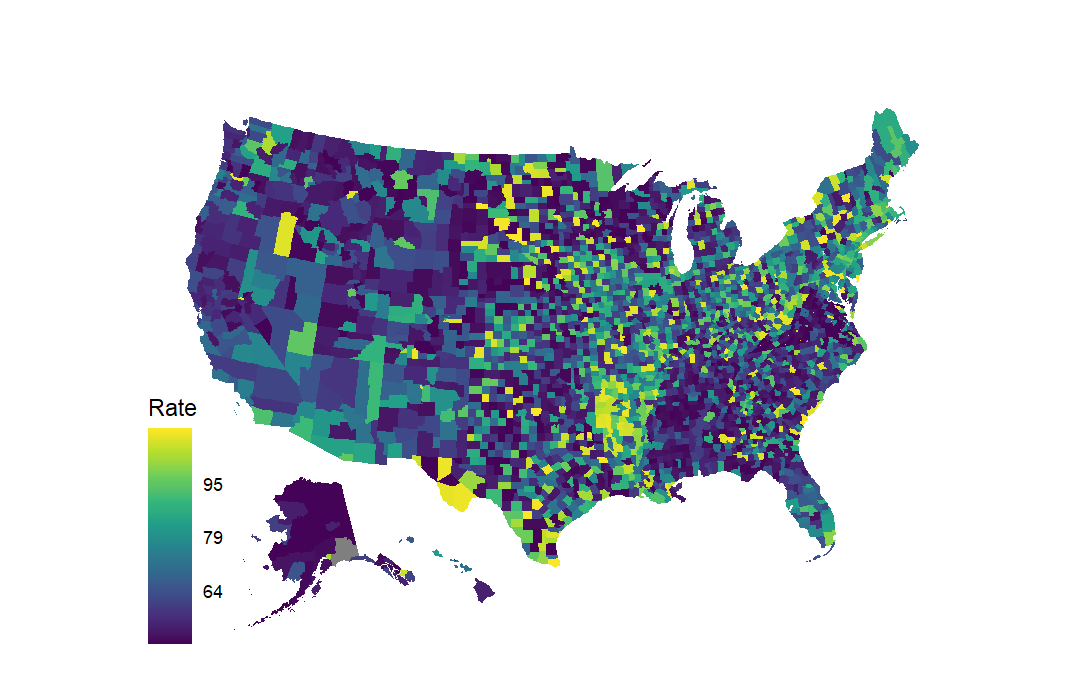

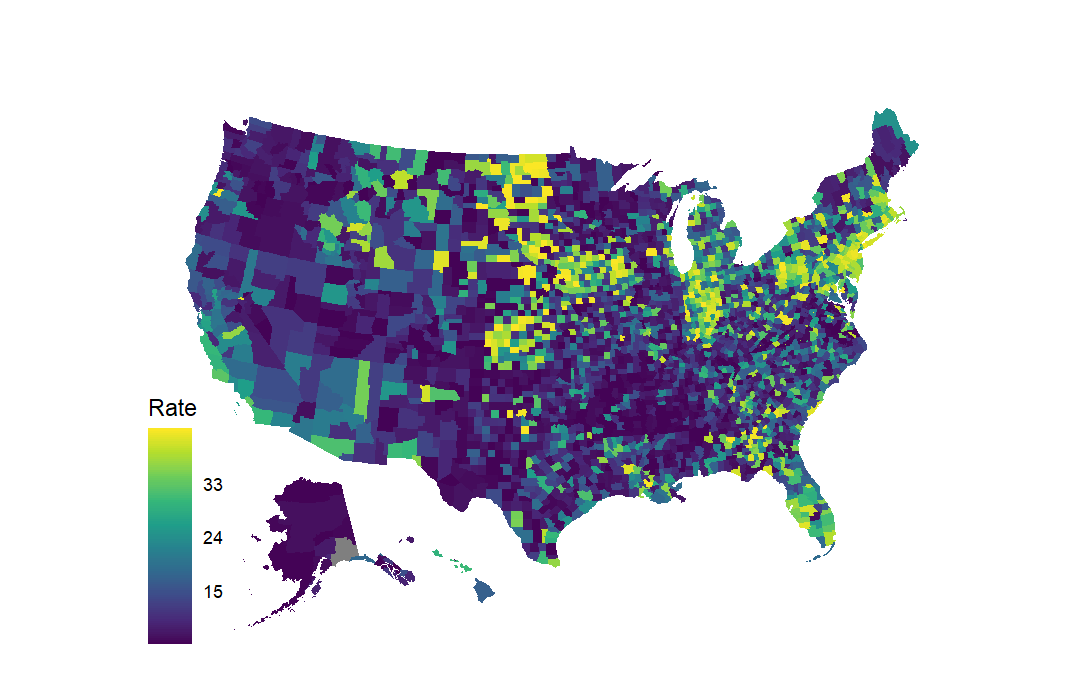
: The scale of the two maps is different and they should not be directly compared. The dark colors indicate less stocking of vaccines per capita. The numbers on the scale show the population-weighted median and interquartile range.

**Table S4.** Descriptive statistics across 3,142 counties

|  | **Mean** | **Range**  **(Min, Max)** | **Median** | **SD** |
| --- | --- | --- | --- | --- |
| **Population count** |  |  |  |  |
| *Total* | 103,341.12 | 66 - 10,081,570 | 25,740 | 331,170.09 |
| $\leq$ *18 years old* | 24,740.76 | 1 - 2,346,589 | 25,740 | 80,052.75 |
| *10-19 years old* | 13,794.05 | 0 - 1,274,790 | 25,740 | 43,617.69 |
| **Overall SVI score** | 50.00 | 0 - 100 | 25,740 | 28.88 |
| **SVI subscale** |  |  |  |  |
| *Socioeconomic status* | 50.00 | 0 – 100 | 50 | 28.88 |
| *Household composition and disability* | 49.99 | 0 – 100 | 50 | 28.88 |
| *Minority status and language* | 49.99 | 0 – 100 | 50 | 28.88 |
| *Housing type & transportation* | 50.00 | 0 – 100 | 50 | 28.88 |
| **Board certified pediatricians** |  |  |  |  |
| *Total* | 15.30 | 0 - 1,439 | 1 | 61.70 |
| *Per 10,000 adolescents^††^* | 2.63 | 0 – 481 | 1 | 9.31 |
| *With additional specialty* | 2.35 | 0 – 204 | 0 | 10.28 |
| *With additional specialty per 10,000 adolescents^††^* | 0.50 | 0 – 60 | 0 | 1.76 |
| Number of physicians certified in pediatrics per 10,000 children^†^ | 3.12 | 0 – 541 | 2 | 10.63 |
|  |  |  |  |  |
| Primary care providers per 10,000 residents^†††^ | 5.20 | 0 – 59 | 5 | 3.78 |
|  |  |  |  |  |
| Percent of children with Medicaid or other means tested health insurance^††^ | 41.27 | 0 – 98 | 41 | 14.11 |
|  |  |  |  |  |
| Percent of children without health insurance^††^ | 6.12 | 0 – 56 | 5 | 5.01 |
| **Urban influence code** |  |  |  |  |
| *Metropolitan* | 0.37 | 0 – 1 | 0 | 0.48 |
| *Micropolitan* | 0.41 | 0 – 1 | 0 | 0.49 |
| *Rural* | 0.22 | 0 – 1 | 0 | 0.41 |
| **DoD Presence** |  |  |  |  |
| *No presence* | 0.90 | 0 – 1 | 1 | 0.30 |
| *Minor base (no health care, or clinic only)* | 0.08 | 0 – 1 | 0 | 0.26 |
| *Major base (hospital or medical center)* | 0.02 | 0 – 1 | 0 | 0.15 |
| *Basic training base* | 0.00 | 0 – 1 | 0 | 0.06 |
| **Tribal presence** |  |  |  |  |
| *No presence* | 0.78 | 0 – 1 | 1 | 0.42 |
| *Lands only without health care* | 0.12 | 0 – 1 | 0 | 0.32 |
| *Non-IHS health care* | 0.08 | 0 – 1 | 0 | 0.28 |
| *Indian Health Service facilities* | 0.02 | 0 – 1 | 0 | 0.15 |
| **State requirement** |  |  |  |  |
| *MenACWY: Aged 11-12* | 0.71 | 0 – 1 | 1 | 0.46 |
| *MenACWY: Age 16* | 0.55 | 0 – 1 | 1 | 0.50 |
| **State recommendation** |  |  |  |  |
| *MenACWY: Ages 11-12* | 0.05 | 0 – 1 | 0 | 0.22 |
| *MenACWY: Age 16* | 0.09 | 0 – 1 | 0 | 0.28 |
| *MenB: Age 16* | 0.11 | 0 – 1 | 0 | 0.31 |
| **State universal purchase policy** |  |  |  |  |
| *MenACWY* | 0.07 | 0 – 1 | 0 | 0.26 |
| *MenB* | 0.07 | 0 – 1 | 0 | 0.26 |

* Within this table, the data are presented as per 1,000; however, they are reported per 100 within the text.

^†^ Denominator = Population count: 10-19 years old

^††^ Denominator = Population count: ≤18 years old

^†††^ Denominator = Primary care providers per 10,000 residen

**Table S5.** Key regression results for MenACWY stocking per capita, private and public*

|  | **MenACWY Stocking per Capita (Private)** | | **MenACWY Stocking per Capita (Public)** | |
| --- | --- | --- | --- | --- |
| **Predictor​** | **Risk-Ratio​** | **95% CI​** | **Risk-Ratio​** | **95% CI​** |
| SVI subscale, socioeconomic status | 1.06 | 1.03, 1.10 | 1.04 | 1.01, 1.07 |
| MenACWY, Universal Purchasing states | -- | -- | 0.61 | 0.40, 0.87 |
| SVI subscale, socioeconomic status + Universal Purchasing Policy for MenACWY | 1.16 | 1.07, 1.26 | -- | -- |
| State Recommendation for MenB, age 16 | 0.55 | 0.40, 0.74 | 0.35 | 0.25, 0.48 |
| SVI subscale, minority status and language | 0.97 | 0.95,1.00 | 0.96 | 0.93, 0.98 |
| Total pediatricians per 10,000 (tertile 2 vs tertile 1) | 0.70 | 0.62, 0.79 | 0.72 | 0.65, 0.80 |
| Total pediatricians per 10,000 (tertile 3 vs tertile 1) | 0.60 | 0.53, 0.69 | 0.70 | 0.62, 0.78 |
| Primary care provider per 10,000 residents rate (tertile 3 vs tertile 1) | 0.84 | 0.74, 0.96 | 0.87 | 0.78, 0.98 |
| Rural County | 1.29 | 1.08, 1.51 | 1.26 | 1.09, 1.46 |
| DoD Basic Training Program (regardless of health care facilities) | 3.03 | 1.42, 5.71 | -- | -- |
| Tribal Lands with Health Care Facility | 0.81 | 0.66, 0.98 | 0.92* | 0.78, 1.10 |
| State Requirement for MenACWY, age 11-12 | -- | -- | 0.74 | 0.53, 0.99 |
| State Recommendation for MenACWY, age 11-12 | 8.70 | 4.15, 16.23 | 2.57 | 1.24, 4.82 |
| State Requirement for MenACWY, age 16 | -- | -- | 1.38 | 1.02, 1.84 |
| State Recommendation for MenACWY, age 16 | 0.42 | 0.25, 0.67 | -- | -- |

* Reporting only significant variables.

Note for interpreting results: A proportion greater than 0.5 would indicate an imbalance in stocking where there were more MenACWY vaccines than MenB vaccines. A proportion of 1 would indicate that a county had only MenACWY vaccines stocked.

References

1. Centers for Disease Control and Prevention (CDC). About Vaccines for Children (VCF). Last reviewed: February 18, 2016. Accessed July 27, 2023. <https://www.cdc.gov/vaccines/programs/vfc/about/index.html>
2. CDC. ACIP Shared Clinical Decision-Making Recommendations. Last reviewed: February 10, 2020. Accessed July 19, 2023.<https://www.cdc.gov/vaccines/acip/acip-scdm-faqs.html>
3. CDC. Estimated routine vaccination coverage (≥1 HPV vaccine dose, ≥1 Tdap, ≥1 MenACWY) among adolescents by age and birth year, National Immunization Survey-Teen, 2015-2021. Last reviewed: September 1, 2022. Accessed: June 26, 2023. <https://www.cdc.gov/vaccines/imz-managers/coverage/teenvaxview/pubs-presentations/nis-teen-vac-coverage-estimates-2015-2021.html>
4. Flanagan BE, Hallisey EJ, Adams E, Lavery A. Measuring Community Vulnerability to Natural and Anthropogenic Hazards: The Centers for Disease Control and Prevention's Social Vulnerability Index. J Environ Health. 2018 Jun;80(10):34-36. PMID: 32327766; PMCID: PMC7179070.
5. Alabama Department of Public Health (ADPH). Immunization. Accessed January 25, 2023. <https://www.alabamapublichealth.gov/immunization/>
6. Alaska Department of Health and Social Services (DHSS). Accessed: January 25, 2023. <https://dhss.alaska.gov/pages/search.aspx?q=meningococcal%20vaccination%20policy>
7. Arizona Department of Health Services (DHS). Arizona Immunization Schedule. Accessed: January 25, 2023. <https://www.azdhs.gov/preparedness/epidemiology-disease-control/immunization/index.php#college-info>
8. Arkansas Department of Health. Immunizations. Accessed: January 25, 2023. <https://www.healthy.arkansas.gov/programs-services/topics/immunizations>
9. California Department of Public Health (CDPH). Meningococcal Disease. Accessed: January 25, 2023. <https://www.cdph.ca.gov/Programs/CID/DCDC/Pages/Immunization/meningococcal.aspx>
10. Colorado Department of Public Health and Environment (CDPHE). School-required Vaccines. Accessed: January 25, 2023. <https://cdphe.colorado.gov/schoolrequiredvaccines>
11. Connecticut State Department of Public Health (DPH). Meningoccocal Disease Information. Accessed: January 25, 2023. <https://portal.ct.gov/DPH/Immunizations/Meningococcal-Disease-Information>
12. Delaware Department of Education (DOE). Delaware Immunization Regulation. Accessed: January 25, 2023. <https://www.doe.k12.de.us/Page/2871>
13. Florida Health. Meningococcal Disease. Accessed: January 25, 2023. <https://www.floridahealth.gov/diseases-and-conditions/meningococcal-disease/index.html>
14. Georgia Department of Public Health (DPH). Immunizations. Accessed: January 25, 2023. <https://dph.georgia.gov/immunizations>
15. Hawaii Department of Health – Disease Outbreak Control Division. School Health Requirements. Accessed: January 25, 2023. <https://health.hawaii.gov/docd/>
16. Idaho Department of Health and Welfare. Child and Adolescent Immunization. Accessed: January 25, 2023. <https://healthandwelfare.idaho.gov/services-programs/children-families/child-and-adolescent-immunization>
17. Illinois Department of Public Health (IDPH). Minimum Immunization Requirements for Children Enrolling or Entering a Child Care Facility or School in Illinois, 2023-2024. Accessed: January 25, 2023. <https://dph.illinois.gov/topics-services/prevention-wellness/immunization/minimum-immunization-requirements.html>
18. Indiana Department of Education (DOE). Immunization Requirements. Accessed: December 18, 2023. <https://www.in.gov/health/immunization/files/2023-24-School-Immunization-English-Requirements-November-2022.pdf>
19. Iowa Health and Human Services (HHS). Child and Adolescent Immunization Schedule by Age. Accessed: December 18, 2023. <https://www.cdc.gov/vaccines/schedules/hcp/imz/child-adolescent.html>
20. Kansas Department of Health and Environment (KDHE) – Division of Public Health. Immunization Requirements. Accessed: January 25, 2023. <https://www.kdhe.ks.gov/324/Immunization-Requirements>
21. Kentucky General Assembly. Title 902 | Chapter 002 | Regulation 060. Accessed: January 25, 2023. <https://apps.legislature.ky.gov/law/kar/titles/902/002/060/>
22. Louisiana Department of Health (LDH). Child Vaccines. Accessed: January 25, 2023. <https://ldh.la.gov/page/toolkit>
23. State of Maine Department of Health and Human Services (DHHS). Maine School Immunization Requirements. Accessed: January 25, 2023. <https://www.maine.gov/dhhs/mecdc/infectious-disease/immunization/documents/ME%20Immunization%20Requirements%20for%20Schools.pdf>
24. Maryland Department of Health – Center for Immunization. Recommended Childhood and Adult Immunization Schedule. Accessed: December 18, 2023. <https://health.maryland.gov/phpa/OIDEOR/IMMUN/Pages/childhood-adult-immunization-schedule.aspx>
25. Massachusetts Department of Public Health. Massachusetts School Immunization Requirements 2023-2024. Accessed: December 18, 2023. <https://www.mass.gov/doc/immunization-requirements-for-school-entry-0/download>
26. CDC. 2023 Recommended Immunizations for Children 7–18 Years Old. Accessed: January 25, 2023. <https://www.cdc.gov/vaccines/schedules/downloads/teen/parent-version-schedule-7-18yrs.pdf>
27. Minnesota Department of Health. Immunizations. Accessed: January 25, 2023. <https://www.health.state.mn.us/people/immunize/basics/readykidswhento.pdf>
28. Mississippi State Department of Health. Mississippi School Immunization Requirements. Accessed: January 25, 2023. <https://msdh.ms.gov/msdhsite/_static/resources/2029.pdf>
29. Missouri Department of Health and Senior Services. 2023-2024 Missouri School Immunization Requirements. Accessed: January 25, 2023. <https://health.mo.gov/living/wellness/immunizations/pdf/2024-school-requirements.pdf>
30. Montana Department of Health and Human Services. Vaccines Required for School Attendance, Preschool -12th Grade. Accessed: January 25, 2023. <https://www.dphhs.mt.gov/assets/publichealth/Immunization/SchoolVaccineRequirementsAugust2019.pdf?ver=2020-01-29-160229-620>
31. Nebraska Department of Health and Human Services. Childhood Immunization Schedule. Accessed: December 18, 2023. <https://dhhs.ne.gov/Immunization/13-Chapter-4-Licensed-Childcare-Rules-and-Regs.pdf>
32. Nevada Department of Health and Human Services – Division of Public and Behavioral Health. Technical Bulletin (Signed). Accessed: January 25, 2023. <https://dpbh.nv.gov/uploadedFiles/dpbhnvgov/content/Programs/SIP/Docs/MCV4%20NEW%207th%20Grade%20Requirement%20TB_signed.pdf>
33. State of New Hampshire Department of Health and Human Services – Division of Public Health Services, Bureau of Infectious Disease Control. School Immunization Requirements 2022-2023. Accessed: January 23, 2023. <https://www.dhhs.nh.gov/sites/g/files/ehbemt476/files/documents2/imm-req-schools22-23.pdf>
34. State of New Jersey Department of Health. K-12 Immunization Requirements. Accessed: January 25, 2023. <https://nj.gov/health/cd/documents/imm_requirements/k12_parents.pdf>
35. New Mexico Department of Health. New Mexico Childcare/Pre-School/School Entry Immunization Requirements (2023-2024). Accessed: January 25, 2023. <https://www.nmhealth.org/publication/view/policy/455/>
36. New York State Department of Health. Meningococcal Disease Fact Sheet. Accessed: December 18, 2023. <https://www.health.ny.gov/publications/2168/>
37. North Carolina Department of Health and Human Services (NCDHHS). Meningococcal Disease. Accessed: January 25, 2023. <https://immunization.dph.ncdhhs.gov/family/vaccines/meningococcal.htm>
38. North Dakota Department of Health and Human Services. 2022-2023 School Immunization Requirements. Accessed: January 25, 2023. <https://www.hhs.nd.gov/sites/www/files/documents/DOH%20Legacy/School%20Requirements%2022-23.pdf>
39. Ohio Department of Health. 2016-2017 Ohio School Entry Requirement for Meningococcal Vaccine. Accessed: January 25, 2023. <https://odh.ohio.gov/wps/wcm/connect/gov/8edd9498-0edc-47ad-86c0-947488ac97c0/2016-2017-Ohio-School-Entry-Requirement-for-Meningococcal-Vaccine.pdf?MOD=AJPERES&CONVERT_TO=url&CACHEID=ROOTWORKSPACE.Z18_M1HGGIK0N0JO00QO9DDDDM3000-8edd9498-0edc-47ad-86c0-947488ac97c0-nLPRHWp>
40. Oklahoma State Department of Health. Vaccines by Age Group. Accessed: December 18, 2023. <https://oklahoma.gov/health/services/personal-health/immunizations/imm-vaccines-by-age-group.html>
41. Oregon Health Authority – Public Health Division. Immunization. Accessed: January 25, 2023. <https://www.oregon.gov/oha/PH/PREVENTIONWELLNESS/VACCINESIMMUNIZATION/GETTINGIMMUNIZED/Documents/SchFlyerEng22.pdf>
42. Pennsylvania Department of Health. School Vaccination Requirements for Attendance in Pennsylvania Schools. Accessed: January 25, 2023. <https://www.health.pa.gov/topics/Documents/School%20Health/SIR8.pdf>
43. State of Rhode Island Department of Health. Immunization and Communicable Disease Testing in Preschool, School, Colleges or Universities (216-RICR-30-05-3). Accessed: January 25, 2023. <https://rules.sos.ri.gov/regulations/part/216-30-05-3>
44. South Carolina Department of Health and Environmental Control (DHEC). Immunization Requirements for Childcare and School. Accessed: January 25, 2023. <https://scdhec.gov/sites/default/files/media/document/Vaccination-Requirements-School-Law-Letter-2022-2023-Revised-2022-08-30.pdf>
45. South Dakota Department of Health. Immunizations Required for SD School Entry. Accessed: December 18, 2023. <https://doh.sd.gov/topics/immunizations-vaccinations/immunizations-required-for-sd-school-entry/>
46. Tennessee Department of Health. Childcare – 12th Grade Immunization Requirements. Accessed: January 25, 2023. <https://www.tn.gov/health/cedep/immunization-program/ip/immunization-requirements/childcare-12th-grade-immunization-requirements.html>
47. Texas Department of State Health Services. 2022 - 2023 Texas Minimum State Vaccine Requirements for Students Grades K-12. Accessed: January 25, 2023. <https://www.dshs.texas.gov/sites/default/files/immunize/school/pdf/6-14-2022-2023-MinReq_K-12.pdf>
48. Utah Department of Health and Human Services. School Childcare Immunization Requirements. Accessed: December 18, 2023. <https://immunize.utah.gov/school-childcare-immunization-requirements/>
49. Vermont Department of Health. School Year 2023-24 Immunization Entry Requirements. Accessed: January 25, 2023. <https://www.healthvermont.gov/sites/default/files/documents/pdf/ID_IZ_K12_schoolentry_iz_requirements.pdf>
50. Virginia Department of Health. School and Day Care Minimum Immunization Requirements. Accessed: January 25, 2023. <https://www.vdh.virginia.gov/immunization/requirements/>
51. Washington State Department of Health. For Preteens and Teens (7-18 Years). Accessed: January 25, 2023. <https://doh.wa.gov/you-and-your-family/immunization/preteens-and-teens>
52. West Virginia Office of Epidemiology and Prevention Services. West Virginia Immunization Requirements for 7th & 12th Graders. Accessed: January 25, 2023. <https://oeps.wv.gov/immunizations/Documents/school/7-12_School_Entry.pdf>
53. Wisconsin Department of Health Services. Wisconsin Student Immunization Law: What schools need to know for Fall 2022. Accessed: January 25, 2023. <https://www.dhs.wisconsin.gov/immunization/wisconsin-student-immunization-law.pdf>
54. Wyoming Department of Health. Immunization Schedules 7 to 18 years old in English. Accessed: January 25, 2023. <https://health.wyo.gov/healthcarefin/chip/immunization-schedules-7-18-years-old-english/>
55. New York Times. Comparing Military Hospitals. Published September 1, 2014. Accessed July 31, 2023. https://www.nytimes.com/interactive/2014/09/01/us/comparing-military-hospitals.html
56. American Board of Pediatrics, Pediatric Physicians Workforce Data Book, 2020-2021, Chapel Hill, NC: American Board of Pediatrics, 2021. Accessed June 20, 2023. <https://www.abp.org/sites/public/files/pdf/workforcedata2020-2021.pdf>
57. Long JC, Delamater PL, Holmes GM. Which definition of rurality should I use? The relative performance of 8 federal rural definitions in identifying rural-urban disparities. Medical Care. 2021;59(10 Suppl 5):S413.
